# The Relation between Hepatotoxicity and the Total Coumarin Intake from Traditional Japanese Medicines Containing Cinnamon Bark

**DOI:** 10.3389/fphar.2016.00174

**Published:** 2016-06-20

**Authors:** Naohiro Iwata, Mosaburo Kainuma, Daisuke Kobayashi, Toshio Kubota, Naoko Sugawara, Aiko Uchida, Sahoko Ozono, Yuki Yamamuro, Norihiro Furusyo, Koso Ueda, Eiichi Tahara, Takao Shimazoe

**Affiliations:** ^1^Department of Clinical Pharmacy and Pharmaceutical Care, Graduate School of Pharmaceutical Sciences, Kyushu UniversityFukuoka, Japan; ^2^Community Medicine Education Unit, Graduate School of Medical Sciences, Kyushu UniversityFukuoka, Japan; ^3^Department of General Internal Medicine, Kyushu University HospitalFukuoka, Japan; ^4^Department of Pediatrics, Matsuyama Red Cross HospitalMatsuyama, Japan; ^5^Department of Japanese Oriental (Kampo) Medicine, Oriental Medical Center, Iizuka HospitalIizuka, Japan

**Keywords:** coumarin, traditional Japanese herbal medicine, hepatotoxicity, tolerable daily intake, cinnamon bark, high performance liquid chromatography, keishibukuryogankayokuinin

## Abstract

Cinnamon bark is commonly used in traditional Japanese herbal medicines (Kampo medicines). The coumarin contained in cinnamon is known to be hepatotoxic, and a tolerable daily intake (TDI) of 0.1 mg/kg/day, has been quantified and used in Europe to insure safety. Risk assessments for hepatotoxicity by the cinnamon contained in foods have been reported. However, no such assessment of cinnamon bark has been reported and the coumarin content of Kampo medicines derived from cinnamon bark is not yet known. To assess the risk for hepatotoxicity by Kampo medicines, we evaluated the daily coumarin intake of patients who were prescribed Kampo medicines and investigated the relation between hepatotoxicity and the coumarin intake. The clinical data of 129 outpatients (18 male and 111 female, median age 58 years) who had been prescribed keishibukuryogankayokuinin (TJ-125) between April 2008 and March 2013 was retrospectively investigated. Concurrent Kampo medicines and liver function were also surveyed. In addition to TJ-125, the patients took some of the other 32 Kampo preparations and 22 decoctions that include cinnamon bark. The coumarin content of these Kampo medicines was determined by high performance liquid chromatography (HPLC). TJ-125 had the highest daily content of coumarin (5.63 mg/day), calculated from the daily cinnamon bark dosage reported in the information leaflet inserted in each package of Kampo medicine. The coumarin content in 1g cinnamon bark decoction was 3.0 mg. The daily coumarin intake of the patients was 0.113 (0.049–0.541) mg/kg/day, with 98 patients (76.0%) exceeding the TDI. Twenty-three patients had an abnormal change in liver function test value, but no significant difference was found in the incidence of abnormal change between the group consuming less than the TDI value (6/31, 19.4%) and the group consuming equal to or greater than the TDI value (17/98, 17.3%). In addition, no abnormal change related to cinnamon bark was found for individual patients. This paper was done to assess the risk of hepatotoxicity by the coumarin contained in Kampo medicines and to clarify whether or not the Kampo preparations in general use that contain cinnamon bark may be safely used in clinical practice.

## Introduction

Cinnamon bark is commonly used in traditional Japanese herbal medicines (Kampo medicines), especially for the treatment of fever and hot flashes. The coumarin contained in cinnamon was used as a flavoring ingredient in foods (Egan et al., 1990), but it was banned from use in food by the Food and Drug Administration (FDA) of the United States in 1954. The FDA made the decision based on data from animal studies that used rats (Egan et al., 1990). A study by Hazleton et al. (1956) indicated that coumarin is hepatotoxic. Since the 1970s, several studies have reported that coumarin improves lymphedema (Casley-Smith et al., 1993a,b), but coumarin related hepatotoxic action was observed in a clinical study by Loprinzi et al. (1999). Some papers have reported that cinnamon improves glucose and lipid metabolism (Khan et al., 2003; Sato et al., 2006), and cinnamon is used as a dietary supplement.

The German Federal Institute for Risk Assessment (BfR) established a tolerable daily intake (TDI) value of 0.1 mg coumarin per kg body weight [BfR (Federal Institute for Risk Assessment), 2006]. Some reports have determined the coumarin content of foods and assessed its toxicity (Sproll et al., 2008; Abraham et al., 2010). It is possible that heavy consumption of the cinnamon included in cookies, tea, and other cinnamon rich foods could exceed the TDI, making it important to pay careful attention to the amount of cinnamon taken in food (Abraham et al., 2010). A 2015 report was published of a case in which a patient, after administration of the cholesterol-lowering drug rosuvastatin, experienced acute hepatitis after taking a cinnamon supplement (Brancheau et al., 2015).

The main pathway of coumarin metabolism is 7-hydroxylation leading to detoxification, and the minor pathway is the metabolism of the lactone ring to form a coumarin 3,4-epoxide intermediate, which can lead to hepatotoxicity (Lake et al., 1989, 1994). 7-hydroxylation, which is predominant in humans, is metabolized by CYP2A6 (Shilling et al., 1969; Cholerton et al., 1992; Pearce et al., 1992; Pelkonen et al., 2000). The clinical data of patients treated with coumarin showed that a few percent were sensitive to the hepatotoxic action (Abraham et al., 2010). It is assumed that the cause of this higher susceptibility is the genetic polymorphism of CYP2A6 with deficient 7-hydroxylation of coumarin, which possibly leads to an increased formation of 3,4-coumarin epoxide. The frequency of poor metabolizers of CYP2A6 in Asian populations is much higher than that of Caucasian populations (Peamkrasatam et al., 2006). Unfortunately, the association between hepatotoxicity and CYP2A6 genetic polymorphism has not been investigated. In addition, the underlying mechanism(s) of human coumarin-related hepatotoxicity has not been elucidated (Abraham et al., 2010).

Cinnamon is generally divided into two types, Ceylon cinnamon and cassia cinnamon. The coumarin content of cassia cinnamon is higher than that of Ceylon cinnamon (Wang et al., 2013). Cassia cinnamon bark is used in traditional Japanese herbal medicines (Kampo medicines). Kampo medicines are of two types; one is a preparation composed of the powdered extract of the herbs specific to each medicine and the other is made by decoction, the extraction of the water-soluble substances by boiling the herbs. In general, side effects are rarely caused by Kampo medicines, but serious hepatotoxicity has been reported (Teschke et al., 2015). It is possible that Kampo medicines that contain coumarin cause hepatotoxicity, however, their coumarin content has not been clarified. Furthermore, the risk of hepatotoxicity from coumarin contained in Kampo medicines has not been assessed in clinical practice.

The Kampo preparations keishibukuryogan (TJ-25) and keishibukuryogankayokuinin (TJ-125) are generally used in Japan as an alternative for the coumarin derivative warfarin. One of the reasons is that they are manufactured by extraction from cinnamon bark. Keishibukuryogan inhibited platelet aggregation in guinea pig whole blood has been reported (Terawaki et al., 2015). In clinical studies, keishibukuryogan was reported to lower blood viscosity, to improve intra-vascular erythrocyte aggregation (Itoh et al., 1992), and to improve the symptoms of patients with non-specific complaints associated with varicose veins of the lower extremities (Hayashi et al., 2014). TJ-125 is a Kampo preparation that includes the extract of coix seeds. In clinical practice, we use TJ-125 more frequently than TJ-25 as alternative for warfarin because TJ-125 contains more cinnamon bark than TJ-25. For these reasons, we focused on patients who had been prescribed TJ-125.

The aims of this study were to evaluate the coumarin content of Kampo medicines derived from cinnamon bark and to assess the risk for hepatotoxicity from the use of these medicines in clinical practice. We developed a system for quantitatively determining the coumarin content of Kampo medicines that uses high performance liquid chromatography (HPLC) to determine the risk of hepatotoxicity for patients prescribed TJ-125. Based on the coumarin content of the Kampo medicines prescribed, we determined the daily intake of coumarin by patients at two hospitals. We investigated the frequency of liver dysfunction during TJ-125 treatment and the possible association between hepatotoxicity and the daily intake of coumarin. The importance of this study is that it clarifies the risk of hepatotoxicity from Kampo medicines derived from cinnamon bark and that it will contribute to safer, more effective use of Kampo medicines.

## Materials and Methods

### Subjects

From April 2008 to March 2013, 351 Japanese outpatients were prescribed TJ-125 at the Kampo Medicine Clinic of Kyushu University Hospital or Iizuka Hospital. The inclusion criteria were as follows: (1) no previous history of liver disease; (2) the body weight was recorded; and (3) examination of liver function was performed before and after the prescription of TJ-125. After exclusions, the data of 129 outpatients was available for analysis. The study was approved by the Ethics Committees of Kyushu University Hospital and Iizuka Hospital.

### Clinical Data Collection

We retrospectively investigated the clinical data of the enrolled outpatients using their electronic medical records. Precise information was obtained on age, sex, body weight, the TJ-125 dosing period, daily dosage, concurrent drugs, and liver function test values: AST, ALT, γ-GTP, ALP, T-Bill, and Alb.

### Assessment of Hepatotoxicity

Adverse events associated with the liver were graded from 0 to 5 by the liver function test values according to Common Terminology Criteria for Adverse Events (CTCAE) version 4.0. Hepatotoxicity was defined as an abnormal change in a liver function test value that was increased at two consecutive testing points during the period of administration of TJ-125. When abnormal change was observed, we surveyed the dosage of the Kampo medicines prescribed and the drug history using the patient’s electronic medical record. The patients’ doctors were contacted for judgments related to the timing of abnormal change. The daily intake of coumarin for each patient was calculated based on the maximum total coumarin content of the prescribed TJ-125 and any concurrent Kampo medicines taken during the dosing period with TJ-125. Patients were classified into two groups: one with less than the TDI value and the other with equal to or greater than the TDI value. To investigate the association between TDI and an abnormal change in liver function, we compared the hepatotoxicity rates of these two groups.

### Materials

Coumarin, acetonitrile (HPLC grade), methanol (HPLC grade), and trifluoroacetic acid were purchased from Wako Pure Chemical Industries (Osaka, Japan).

Twenty-five Kampo preparations made with extract granules were purchased from Tsumura & Co. (Tokyo, Japan): kakkonto (TJ-1), hachimijiogan (TJ-7), saikokeishito (TJ-10), saikokeishikankyoto (TJ-11), saikokaryukotsuboreito (TJ-12), goreisan (TJ-17), keishikajutsubuto (TJ-18), shoseiryuto (TJ-19), keishibukuryogan (TJ-25), keishikaryukotsuboreito (TJ-26), maoto (TJ-27), mokuboito (TJ-36), tokishigya-kukagoshuyushokyoto (TJ-38), ryokeijutsukanto (TJ-39), keishito (TJ-45), juzentaihoto (TJ-48), goshakusan (TJ-63), shakanzoto (TJ-64), nyoshinsan (TJ-67), keishininjinto (TJ-82), jidabokuippo (TJ-89), goshajinkigan (TJ-107), saireito (TJ-114), tokikenchuto (TJ-123) and keishibukuryogankayokuinin (TJ-125). Saikokeishito (N10), saikokeishikankyoto (N11), saikokaryukotsuboreito (N12) and kumibinroto (N311) were purchased from Kotaro Pharmaceutical Co., Ltd. (Osaka, Japan). Keishikaryojutsubuto (SG-18R) was purchased from Ohsugi Pharmaceutical Co., Ltd. (Osaka, Japan). Keishikaogito (TY-026) was purchased from Toyo Yakuko Co., Ltd. (Tokyo, Japan). Kakkonkajutsubuto (S-07) was purchased from Sanwa Shoyaku Co., Ltd. (Tochigi, Japan). Hachimigan was purchased from Uchida Wakanyaku Co., Ltd. (Tokyo, Japan). The cinnamon bark used for decoction was purchased from Tochimoto Tenkaido (Osaka, Japan).

### Quantitative Determination of the Coumarin Used in Kampo Preparations and in Cinnamon Bark

The coumarin content of the Kampo preparations and cinnamon bark was determined using HPLC. The Kampo preparations used were manufactured between 2011 and 2013. The extraction of the components of the Kampo preparations followed the procedures of Okamura et al. (1999). Briefly, 0.5 g of the Kampo preparation was sonicated for 30 min with 30 mL of 65 % methanol, then centrifuged at 1,500 × *g* for 10 min. For the cinnamon bark, 20 g of cinnamon bark was heated for 40 min with 600 mL of water, then centrifuged at 1,500 × *g* for 20 min. The supernatant was filtered through a 0.45 μm syringe filter (Merck Millipore, Darmstadt, Germany) after which 10 μL of solution was injected into the HPLC system.

The samples tested were from three different production lots, when possible. In cases for which it was impossible to obtain Kampo preparations from three production lots, one or two production lots were tested. After confirming that the extraction error was less than 3 %, each sample had a single extraction analyzed in duplicate by HPLC.

The daily dosage was obtained from the information leaflet inserted in the package of each Kampo preparation. The coumarin content in the daily dosage was determined by the mean amount in 1–3 production lots. The coumarin content in the daily dosage of cinnamon bark was determined by quantitation analysis using HPLC. The information in the package states that the standard daily dosage of TJ-125 contains 4 g of cinnamon bark. Therefore, we compared the coumarin content of TJ-125 with 4 g of cinnamon bark for decoction use. We then calculated the coumarin content per 1 g of cinnamon bark contained in the other Kampo medicines.

### HPLC Conditions

The HPLC method used to determine the coumarin content of the different Kampo medicines was modified from that used for the determination of the (E)-cinnamic acid contained in keishibukuryogan extract found in the Japanese Pharmacopoeia 16th Edition (Ministry of Health, Labour and Welfare, Japan, 2011). A Shimadzu LC10A system (Shimadzu Co., Kyoto, Japan) equipped with a photodiode array detector (SPD-M10A_V P_) was used. A Gemini-Nx5u C18 110A column (250 mm × 4.6 mm I.D., 5 μm, Phenomenex, Torrance, CA, USA) was used with a mobile phase that consisted of a mixture of water, acetonitrile, and trifluoroacetic acid (750:250:0.5, v/v/v) delivered at a flow rate of 0.5 mL/min. The column temperature was maintained at 40°C. The absorbance detector was set at 273 nm.

### Calibration Curve

The standard coumarin solutions were prepared to 0.5, 1, 5, 10, 15, and 20 mg/mL in water-acetonitrile solution (1:1, v/v). A calibration curve obtained by plotting the peak area versus the concentration of coumarin, and the quantity of coumarin in the extraction was determined by use of an absolute calibration curve method.

### Statistical Analysis

Data are shown as mean ± SD or median (range). Statistical differences between the group consuming less than the TDI value and the group equal to or greater than the TDI value were evaluated using Fisher’s exact test and Wilcoxon rank sum test, as appropriate. *P*-values <0.05 were considered statistically significant.

## Results

The clinical characteristics of the patients are shown in **Table 1**. The median age of the 18 male and 111 female patients was 58 years. The dosing period was for a median 151 days. In addition to TJ-125, the patients concurrently took other of the 32 Kampo preparations and 22 decoctions (median 1, range 0–3).

**Table 1:** Clinical characteristics of the participants (*n* = 129).

**Sex**
Male, *n* (%)	18	(14.0)
Female, *n* (%)	111	(86.0)
Age (years)	58	(21–91)
Body weight (kg)	55.0	(38.0–88.7)
**Liver function test values (before prescription of TJ-125)**		
AST (IU/L)	20	(11–74)
ALT (IU/L)	15	(5–101)
ALP (IU/L)	208	(80–473)
γ-GTP (IU/L)	18	(8–190)
T-Bill (mg/dL)	0.6	(0.2–2.0)
Alb (g/dL)	4.3	(2.4–5.3)
Dosing period (days)	151	(14–1404)
Concurrent Kampo medicines (*n*)	1	(0–3)
Daily intake of cinnamon bark contained in Kampo medicines (g/day)	5.0	(4.0–16.0)
Daily intake of coumarin (mg/kg/day)	0.113	(0.049–0.541)
More than the TDI of coumarin, *n* (%)	98	(76.0)
Incidence of abnormal change in liver function test values, n (%)	23	(17.8)

*Variables expressed as median (range).*

**FIGURE 1:**
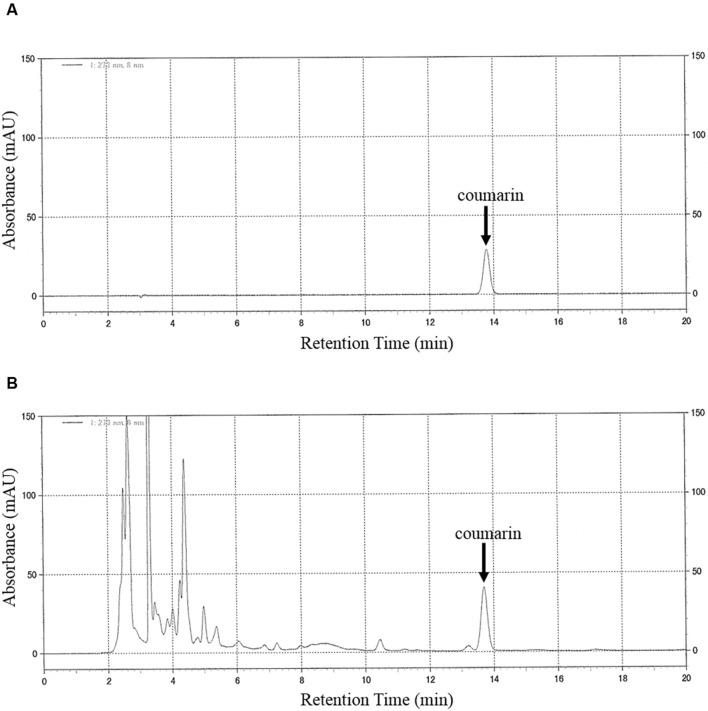
**HPLC chromatograms of (A) coumarin (10 mg/mL, standard) and (B) TJ-125.** Chromatographic peaks were detected at a wavelength of 273 nm. The mobile phase consisted of water, acetonitrile and trifluoroacetic acid (750:250:0.5, v/v/v). The flow rate of the mobile phase was 0.5 ml/min. The injection volume was 10 μL. The column temperature was set at 40°C. The retention time of coumarin was 13.7 min.

The chromatograms of the authentic coumarin (**Figure 1A**) and the extract of TJ-125 (**Figure 1B**) were obtained by HPLC analysis. The peak, at the retention time of 13.7 min for TJ-125 corresponded to that of coumarin. The coumarin content of the TJ-125 was separated from the other ingredients. In addition, the peak for coumarin in the concurrent Kampo medicines and the cinnamon bark, separated from the other ingredients, was determined (data not shown). Excellent linearity was observed over the studied concentration range of the calibration curve, and the correlation coefficient (*r*^2^) was 0.999. With this analytical method, the limit of detection of coumarin was 0.1 mg/mL.

The coumarin content in the daily dosage of each Kampo preparation is shown in **Table 2**. TJ-125 contained 5.63 mg coumarin per day, the highest content among the 33 Kampo preparations. Coumarin was not detected in TJ-63, TJ-107, or TY-026. The mean value for coumarin content as a daily dosage was 1.43 mg ± 0.20. The coumarin content of 4 g of cinnamon bark was 12.1 mg. Cinnamon bark had 2.1 times higher coumarin content than TJ-125. The mean value for coumarin in 1 g of cinnamon bark was 0.50 mg ± 0.06, with TJ-17 having the highest coumarin per g at 1.65 mg.

**Table 2:** The coumarin content of the daily dosage from the information leaflet inserted in the package of each Kampo preparation and for cinnamon bark.

	Cinnamon bark content (g/day)	Coumarin content (mg/day)	Coumarin content (mg/g of cinnamon bark)	*n*
Cinnamon bark	4.0	12.07	3.02	1
Keishibukuryogankayokuinin (TJ-125)	4.0	5.63 ± 0.94	1.41 ± 0.24	3
Mokuboito (TJ-36)	3.0	4.00	1.33	1
Keishibukuryogan (TJ-25)	3.0	2.92 ± 0.09	0.97 ± 0.03	3
Ryokeijutsukanto (TJ-39)	4.0	2.80 ± 0.96	0.70 ± 0.24	3
Goreisan (TJ-17)	1.5	2.48 ± 0.07	1.65 ± 0.05	3
Nyoshinsan (TJ-67)	2.0	2.32	1.16	1
Kakkonto (TJ-1)	2.0	2.00 ± 0.07	1.00 ± 0.03	3
Kakkonkajutsubuto (S-07)	2.0	1.80 ± 0.17	0.90 ± 0.09	3
Saireito (TJ-114)	2.0	1.15	0.57	2
Saikokeishikankyoto (N11)	3.0	1.13	0.38	1
Keishininjinto (TJ-82)	4.0	1.09 ± 0.54	0.27 ± 0.14	3
Saikokeishito (N10)	2.5	1.03	0.41	1
Saikokeishito (TJ-10)	2.0	0.86 ± 0.20	0.43 ± 0.10	3
Saikokeishikankyoto (TJ-11)	3.0	0.79 ± 0.05	0.26 ± 0.02	3
Saikokaryukotsuboreito (TJ-12)	3.0	0.69 ± 0.04	0.23 ± 0.01	3
Saikokaryukotsuboreito (N12)	3.0	0.66	0.22	1
Hachimijiogan (TJ-7)	1.0	0.65 ± 0.22	0.65 ± 0.22	3
Keishikaryojutsubuto (SG-18R)	4.0	0.55 ± 0.14	0.14 ± 0.03	3
Keishikaryukotsuboreito (TJ-26)	4.0	0.51 ± 0.23	0.13 ± 0.06	3
Shoseiryuto (TJ-19)	3.0	0.48 ± 0.22	0.16 ± 0.07	3
Keishikajutsubuto (TJ-18)	4.0	0.46	0.11	2
Maoto (TJ-27)	4.0	0.41 ± 0.03	0.10 ± 0.01	3
Kumibinroto (N311)	3.0	0.36 ± 0.02	0.12 ± 0.01	3
Tokikenchuto (TJ-123)	4.0	0.34 ± 0.26	0.08 ± 0.07	3
Shakanzoto (TJ-64)	3.0	0.26 ± 0.05	0.09 ± 0.02	3
Keishito (TJ-45)	4.0	0.26 ± 0.01	0.06 ± 0.00	3
Juzentaihoto (TJ-48)	3.0	0.24 ± 0.04	0.08 ± 0.01	3
Jidabokuippo (TJ-84)	3.0	0.24 ± 0.04	0.08 ± 0.01	3
Hachimigan	1.0	0.22 ± 0.02	0.22 ± 0.02	3
Tokishigyakukagoshuyushokyoto (TJ-38)	3.0	0.15	0.05	1
Goshakusan (TJ-63)	1.0	ND	ND	2
Goshajinkigan (TJ-107)	1.0	ND	ND	3
Keishikaogito (TY-026)	4.0	ND	ND	3
Mean ± SD		1.43 ± 0.20	0.50 ± 0.06	

*n, number of production lots for HPLC analysis; ND, not detected; The coumarin content and coumarin content included in 1 g of cinnamon bark are shown as mean ± SD for n = 3, mean for n = 2, and the measured value for n = 1*.

The total daily dosage of cinnamon bark when adding that from TJ-125 to that from the concurrent Kampo medicines ranged from 4 to 16 g. The median daily intake of coumarin from the Kampo medicines was 0.113 mg/kg/day, with 98 patients exceeding the TDI (**Table 1**). There were six cases (19.4%) of abnormal change in liver function in the group with less than the TDI value and 17 (17.3%) in the group with equal to or greater than the TDI value (**Table 3**). No significant difference between these groups was found concerning the incidence of abnormal change in liver function. Statistically significant differences were found for body weight, and daily intake of coumarin. The liver function test values before and after the prescription of TJ-125 are also shown in **Table 3**. Before the prescription of TJ-125, γ-GTP was statistically different between the group with less than the TDI value and the group with equal to or greater than the TDI value. None of the highest liver function test values after the prescription of TJ-125 were significantly different between these groups. Among the 23 patients with an abnormal change in liver function, AST, ALT, ALP, γ-GTP, T-Bill, and Alb were related to the poor function of 4, 12, 2, 4, 3, and 7 patients, respectively. Possible causes of these abnormal changes were diseases other than hepatopathy for six patients, concomitant drugs other than the concurrent Kampo medicines for four, life style or age for three, and problems unrelated to the intake period of Kampo medicines for ten. None of the cases was related to cinnamon bark.

**Table 3:** Daily intake of the coumarin contained in Kampo medicines and the occurrence of liver disease.

	Group with less than the TDI (*n* = 31)	Group with equal to or greater than the TDI (*n* = 98)	*P*-value
Sex			
Male, n (%)	5 (16.1)	13 (13.3)	0.767
Female, n (%)	26 (83.9)	85 (86.7)	
Age (years)	56 (27–78)	59 (21–91)	0.362
Body weight (kg)	64 (43–88.7)	53.4 (38–84.8)	<0.0001^∗^
Dosing period (days)	147 (21–869)	152.5 (14–1404)	0.639
Daily intake of coumarin (mg/kg/day)	0.086 (0.049–0.099)	0.124 (0.101–0.541)	<0.0001^∗^
Liver function test values
Before the prescription of TJ-125			
AST (IU/L)	20 (11–54)	19.5 (11–74)	0.493
ALT (IU/L)	17 (8–98)	14.5 (5–101)	0.052
ALP (IU/L)	211 (106–355)	206 (81–473)	0.817
γ-GTP (IU/L)	25 (10–190)	16 (8–102)	0.001^∗^
T-Bill (mg/dL)	0.6 (0.3–1.6)	0.6 (0.2–2.0)	0.876
Alb (g/dL)	4.3 (3.3–4.8)	4.3 (2.4–5.3)	0.827
After the prescription of TJ-125 (highest values)			
AST (IU/L)	19 (13–59)	21 (11–98)	0.586
ALT (IU/L)	18 (8–61)	18 (5–126)	0.610
ALP (IU/L)	208 (109–344)	213.5 (15–486)	0.912
γ-GTP (IU/L)	26 (12–120)	20 (7–511)	0.077
T-Bill (mg/dL)	0.7 (0.3–1.3)	0.7 (0.2–1.8)	0.674
Alb (g/dL)	4.2 (3–4.8)	4.2 (2.2–5.1)	0.666
Incidence of abnormal change in liver function test values, *n* (%)	6 (19.4)	17 (17.3)	0.792

*Variables expressed as median (range). Sex and incidence of abnormal change in liver function test values were evaluated by Fisher’s exact test and the others were evaluated by Wilcoxon rank sum test. ^∗^Significantly different between the group with less than the TDI and the group with equal to or greater than the TDI (P < 0.05)*.

## Discussion

The TDI of coumarin was established in Europe for protection against hepatotoxic events. A survey of foods containing coumarin was conducted to estimate the exposure to coumarin and to assess its hepatotoxicity. By contrast, no reports of the coumarin content of Kampo medicines or risk assessments of hepatotoxicity from the coumarin contained in the Kampo medicines used in clinical practice in Japan have been published. In the present study, we evaluated the coumarin content of the Kampo medicines used in clinical practice in Japan to investigate the association between daily coumarin intake and the occurrence of hepatotoxicity.

The coumarin content of 33 Kampo preparations and cinnamon bark was determined by HPLC. The HPLC methodology for determining the coumarin content of foods and plants has been published (Biavatti et al., 2004; Sproll et al., 2008). However, the coumarin content in Kampo medicines has not been reported. The mobile phase for determining the coumarin content of Asteraceae is acetonitrile/water (1:1, v/v; Biavatti et al., 2004). This method made it difficult to separate the peak of coumarin from other peaks contained in the seven Kampo preparations that contain bupleurum root (TJ-10, TJ-11, TJ-12, TJ-114, N10, N11, and N12). Therefore, the mobile phase in this study was changed to water/acetonitrile/trifluoroacetic acid (750:250:0.5, v/v/v). We were thus able to measure, under the same conditions, the quantity of coumarin contained in a wide range of Kampo preparations.

Of the 33 Kampo preparations surveyed, TJ-125 has the highest content of coumarin per daily dose (5.63 mg/day; **Table 2**). Calculation based on quantitative estimation by HPLC and the coumarin content per daily dosage from the information leaflet inserted in the package of 30 Kampo preparations (excepting TJ-63, 107, and TY-026) were from 0.15 to 5.63 mg. However, the daily cinnamon bark content in the daily dosage from the package insert information varied, ranging from 1 to 4 g. The coumarin content in 1 g of cinnamon bark varied greatly among these Kampo preparations, ranging from 0.05 to 1.65 mg. These results suggest that the coumarin content of the preparations was not related to the amount of cinnamon bark used in their production. Previous research has shown that the coumarin content of cinnamon bark varies by cultivation site (Sagara et al., 1987). It has been reported that there is as much as 21 times difference in the coumarin content of 13 cassia cinnamon varieties from different places of cultivation and that some cinnamons do not contain coumarin (He et al., 2005). Another study concluded that there is a difference in the amount of ephedrine contained in kakkonto and shoseiryuto extract preparations that depends on the method of extraction and manufacturing (Nishioka et al., 1996). In the present study, there is some possibility of influence by differences in the area the cinnamon bark was cultivated, the method of extraction and the manufacturing process of each manufacturer, or interaction of the component galenicals of the Kampo preparations; however, the cause(s) of the differences in the coumarin content of the Kampo preparations studied is unclear.

The TDI of coumarin is 5.0 mg/day for an adult weighing 50 kg when calculated as 0.1 mg/kg body weight. The daily content of coumarin in TJ-125 was 5.63 mg, which made it the only Kampo preparation among the 33 tested that exceeded the TDI. The median daily intake of coumarin of the 129 patients tested was 0.113 (0.049–0.541) mg/kg/day, with 98 having more than 0.1 mg/kg/day: more than 75% of the patients exceeded the TDI. These findings show that patients administered TJ-125 are routinely exceeding the TDI and that the risk of liver injury may be increased when TJ-125 is administrated in combination with other Kampo medicines that contain cinnamon bark. However, no cases of abnormal liver function caused by cinnamon bark were observed. Furthermore, there was no significant difference in the incidence of unusual changes in the liver function test values between the group of patients consuming less than the TDI and the group consuming an amount equal to or greater than the TDI. These results indicate that there is no association between the intake of the coumarin found in Kampo medicines and the occurrence of liver injury.

TJ-125 is a mixture of six constituent herbs: cinnamon bark, moutan bark, Paeonia lactiflora, peach kernel, Poria sclerotium, and coix seed. These herbs are not included in the traditional Chinese herbal medicines that have been reported to cause hepatotoxicity (Teschke et al., 2015). It is known that there are 10 chemical components other than coumarin in TJ-25, and that they are also included in TJ-125 (Seto et al., 2000). No hepatotoxicity by these ingredients has been reported. Moutan bark has been shown to have a protective effect against cytotoxicity in human liver cells (Shon and Nam, 2002). The paeonol in moutan bark protects against epirubicin-induced hepatotoxicity (Wu et al., 2016). Additionally, the extract of *Paeonia lactiflora* and *Astragalus membranaceus* has a protective effect on carbon tetrachloride-induced liver fibrosis in rats and on Bacillus Calmette-Gueìrin and lipopolysaccharide induced liver injury in mice (Sun et al., 2007, 2008). The coumarin contained in cinnamon bark might by itself induce liver disease, but the hepatotoxicity of coumarin may be suppressed by other component galenicals. In order to clarify components that might affect liver function, further animal model experiments using purified compounds are necessary. Our results indicate that consumption of the coumarin contained in the Kampo medicines used in clinical practice that exceeds the TDI has little effect on hepatotoxicity.

A limitation to this study is that none of the patients were prescribed cinnamon bark only. It is possible that cinnamon bark interacts with other herbal medicines. Further studies are needed about the interaction between cinnamon bark and other Kampo medicines that do not contain coumarin. Moreover, the coumarin content of the 22 decoctions was calculated by the coumarin content of the constituent cinnamon bark, but the content may differ greatly when used in combination with other constituents. Also, it has been reported that only a small percentage of the Caucasian population is sensitive to coumarin (Abraham et al., 2010). The coumarin-related hepatotoxic incidence in Japan and the hepatotoxic incidence rate caused by cinnamon bark are not known. Therefore, the number of patients studied, 129, may be too small to provide conclusive results. In addition, because the present study was of retrospective in design, the amount of data available for analysis in the electronic medical record was limited. We estimated the cause of abnormal liver function change by the electronic medical record and by inquiry to the doctor in charge of the patient. However, we were not able to obtain the records on the medication compliance of the patients, medicines purchased over the counter at a pharmacy, or medicines prescribed by other hospitals. These factors may be related to the changes of liver function. There is also a possibility that some cases of liver injury were missed because liver function testing was not routinely performed during follow-up. Finally, this study also showed a bias toward the number of women because TJ-125 is often prescribed to patients with gynecological diseases. Thus further investigation that includes more patients will be required to provide more detailed information.

## Conclusion

Our evaluation of the coumarin content of Kampo medicines derived from cinnamon bark and their risk for hepatotoxicity in clinical practice showed that many of the patients prescribed TJ-125 had coumarin intake exceeding the TDI. However, no cases of abnormal liver function caused by cinnamon bark were observed. Our results suggest that ingestion of the coumarin contained in Kampo medicines that exceeds the TDI is not associated with hepatotoxicity and that Kampo medicines may be safely used in clinical practice, without concern for exceeding the TDI.

## Author Contributions

All authors listed, have made substantial, direct and intellectual contribution to the work, and approved it for publication.

## Conflict of Interest Statement

The authors declare that the research was conducted in the absence of any commercial or financial relationships that could be construed as a potential conflict of interest.

## Abbreviations

Alb: albumin
ALP: alkaline phosphatase
ALT: aspartate aminotransferase
AST: alanine aminotransferase
CTCAE: Common Terminology Criteria for Adverse Events
FDA: Food and Drug Administration
γ-GTP: γ-glutamyl transpeptidase
HPLC: high performance liquid chromatography
T-Bill: total bilirubin
TDI: tolerable daily intake
